# Determination of safety margin of nasal septum osteotomy for sphenoid sinus in cleft lip and palate patients

**DOI:** 10.1186/s12903-024-04361-z

**Published:** 2024-05-27

**Authors:** Ergin Ozturk, Gorkem Tekin, Nesrin Saruhan Kose, Mehmet Ugurlu, Elif Bilgir, Omur Dereci

**Affiliations:** 1 Oral and Maxillofacial Surgeon, Private Practice, Eskişehir, Turkey; 2 Department of Oral and Maxillofacial Surgery, Faculty of Dentistry, Eskişehir Osmangazi University, Eskişehir, Turkey; 3 Department of Orthodontics, Faculty of Dentistry, Eskişehir Osmangazi University, Eskişehir, Turkey; 4 Department of Oral and Maxillofacial Radiology, Faculty of Dentistry, Eskişehir Osmangazi University, Eskişehir, Turkey

**Keywords:** Cleft lip palate, Nasal septum osteotomy, Sphenoid sinus

## Abstract

**Background:**

Nasal septum osteotomy is used for separating the nasal septum and maxilla during a Le Fort I osteotomy. If this osteotomy is applied too high or is tilted into the nasal cavity, the sphenoid sinus and various adjacent vital structures may be damaged, and serious bleeding, neurological complications, blindness or even death may occur. The aim of this study is to determine the safety margin of the nasal septum osteotomy for sphenoid sinus during the Le Fort I surgery in cleft lip and palate (CLP) patients.

**Methods:**

Twenty cleft lip and palate (the CLP group) and 20 healthy individuals (the control group) were included in this study. Three values (two lines and an angle) were measured by cone beam computed tomography (CBCT). The first line is the line passing through the junction of the spina nasalis anterior point and the lower point of the perpendicular lamina of the palatine bone. The undersired line is the line passing through the junction of the spina nasalis anterior point and the lower anterior border of the base of the sphenoid sinus. The osteotomy angle is the angle between these two lines.

**Results:**

In the control group; a surgical line of 44.11–61.14 mm (mean 51.91 ± 4.32), an undesired line of 52.48–69.58 mm (mean 59.14 ± 5.08) and an angle of 18.22–27.270 (mean 22.66 ± 2.55) were found, while in the CLP group, a surgical line of 34.53–51.16 mm (mean 43.38 ± 4.79), an undesired line of 46.86–61.35 mm (mean 55.02 ± 3.24) and an angle of 17.60–28.810 (mean 22.60 ± 2.81) were found.

**Conclusions:**

Although the angle to the sphenoid sinus was not significantly affected by CLP, careful planning and consideration of these anatomical differences are crucial to prevent complications and ensure the safety of Le Fort I surgery in CLP patients. Further research with larger sample sizes and subgroup analysis of unilateral and bilateral CLP cases is needed to improve our understanding of these anatomical variations and improve surgical approaches to individuals with CLP undergoing orthognathic procedures.

**Supplementary Information:**

The online version contains supplementary material available at 10.1186/s12903-024-04361-z.

## Background

Cleft lip and palate (CLP) is a congenital deformity that occurs due to abnormal facial development during pregnancy and are seen in one of every 1000 newborn babies [1]. Treatment plans for CLP patients vary according to age and existing problems [2]. Orthognathic surgery is required to correct dentofacial deformities in one out of every four CLP patients [3, 4].

Le Fort I surgery is a procedure that interacts with the nasal cavity, posterior ethmoid air cells, posterior orbit, lower orbital fissure, pterygopalatine fossa and maxillary sinus, and caution should be exercised in the surgical intervention. The nasal septum osteotomy used during nasal cartilage and vomer separation from the nasal base should be kept slightly inclined downward due to the tendency to deflect upward [5]. There is a risk of direct damage to the sphenoid sinus during Le Fort I surgery. During nasal septum osteotomy, the sphenoid sinus inferior anterior wall can be perforated due to the angulation and location of the nasal septum osteotome, and even the osteotome can penetrate the sphenoid sinus. Some of the complications related to sphenoid sinus damage are: internal carotid artery complications, sphenoid sinusitis, malunion of the vomer bone, and cranial nerve injuries [6, 7]. Vomerosphenoidal disarticulations have also been reported [8]. Because of these complications, we must be careful to avoid sphenoid sinus injuries during surgery.

In our study, we aimed to determine the safety margin of nasal septum osteotomy according to the sphenoid sinus during Le Fort I surgery in CLP patients.

## Methods

### Samples

Our study was planned retrospectively with the approval of Eskişehir Osmangazi University Non-Invasive Clinical Research Ethics Committee (date: 17 December 2019, no: 2019–403) and was carried out in accordance with the ethical standards specified in the 1964 Declaration of Helsinki and its subsequent amendments. Forty (20 CLP and 20 control) individuals who had not undergone prior orthognathic surgery, who had no congenital or acquired craniofacial deformities other than a CLP, who had completed their growth and who were older than 14 years old were included in this study.

The growth and development stages of the patients were determined by evaluating the cervical vertebra maturation on lateral cephalometric radiographs obtained from CBCT images using the Hassel-Farman method [9], and patients who completed their adolescent growth were included in the study. Patients who had previous alveolar cleft repair but required revision were excluded from the study.

### CBCT measurements

Patients were allocated into two groups. In CLP group, unilateral and bilateral CLP patients; and in control group, orthognathic surgery patients without cleft (maxillary deficiency patients). Cone-beam computed tomography (CBCT) imaging was performed for each patient using a CBCT machine, and CBCT images were acquired in the standing position using a CBCT scanner (Planmeca Promax 3D mid, Helsinki, Finland) using the following exposure parameters: 94 kVp tube voltage, 14 mA tube current and 27 s time. CBCT data was transferred to a software program (SYNAPSE version 4.4.000, Fujifilm, Tokyo, Japan) in DICOM format. The FH plane was set parallel to the horizontal plane and head position calibration was performed.

**Fig. 1:**
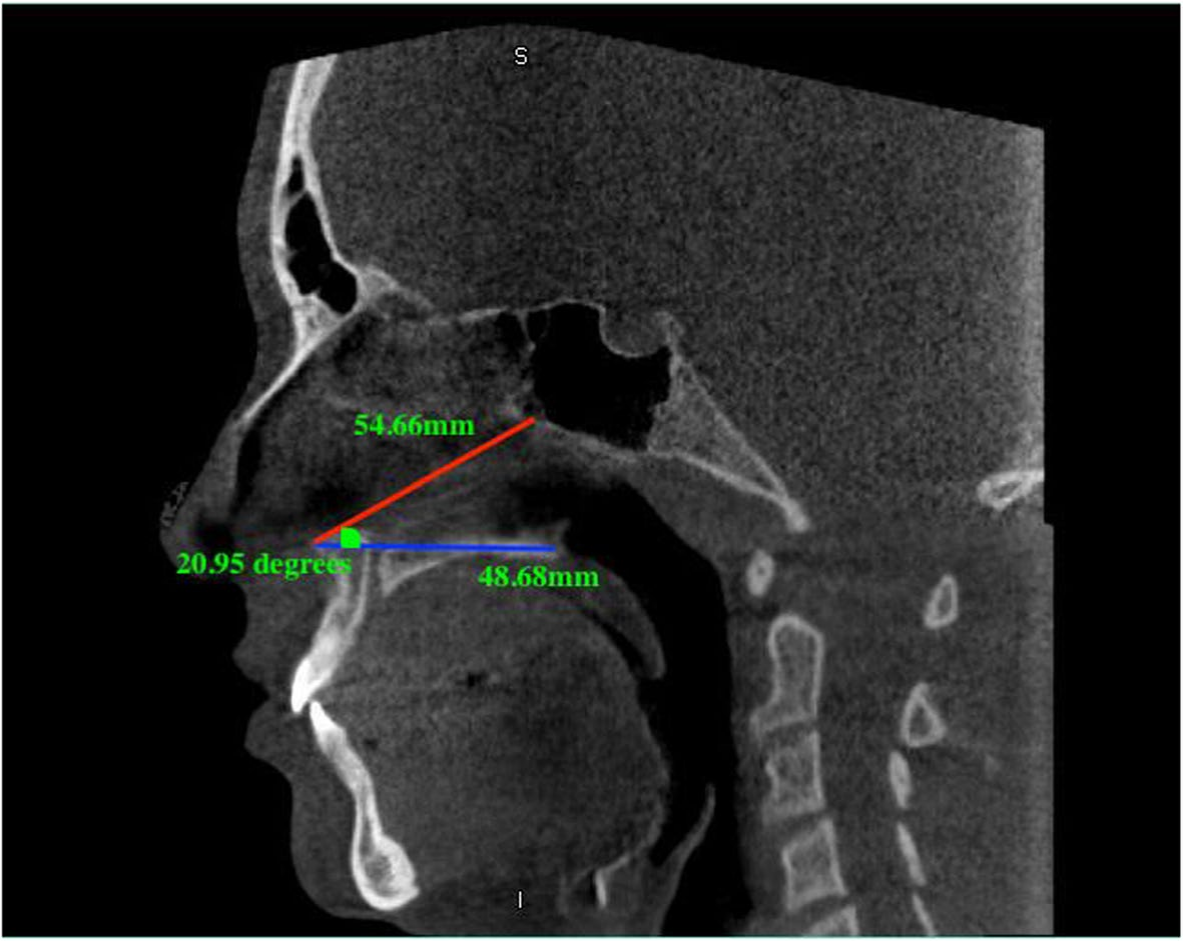
Length and angle measurements in the control group. Blue line; the surgical line extended between the spina nasalis anterior point and the junction of the lower point of the perpendicular layer of the palatine bone, red line; the undesired line extended between the spina nasalis anterior and the lower anterior edge of the sphenoid sinus floor, green angle; between these two lines

**Fig. 2:**
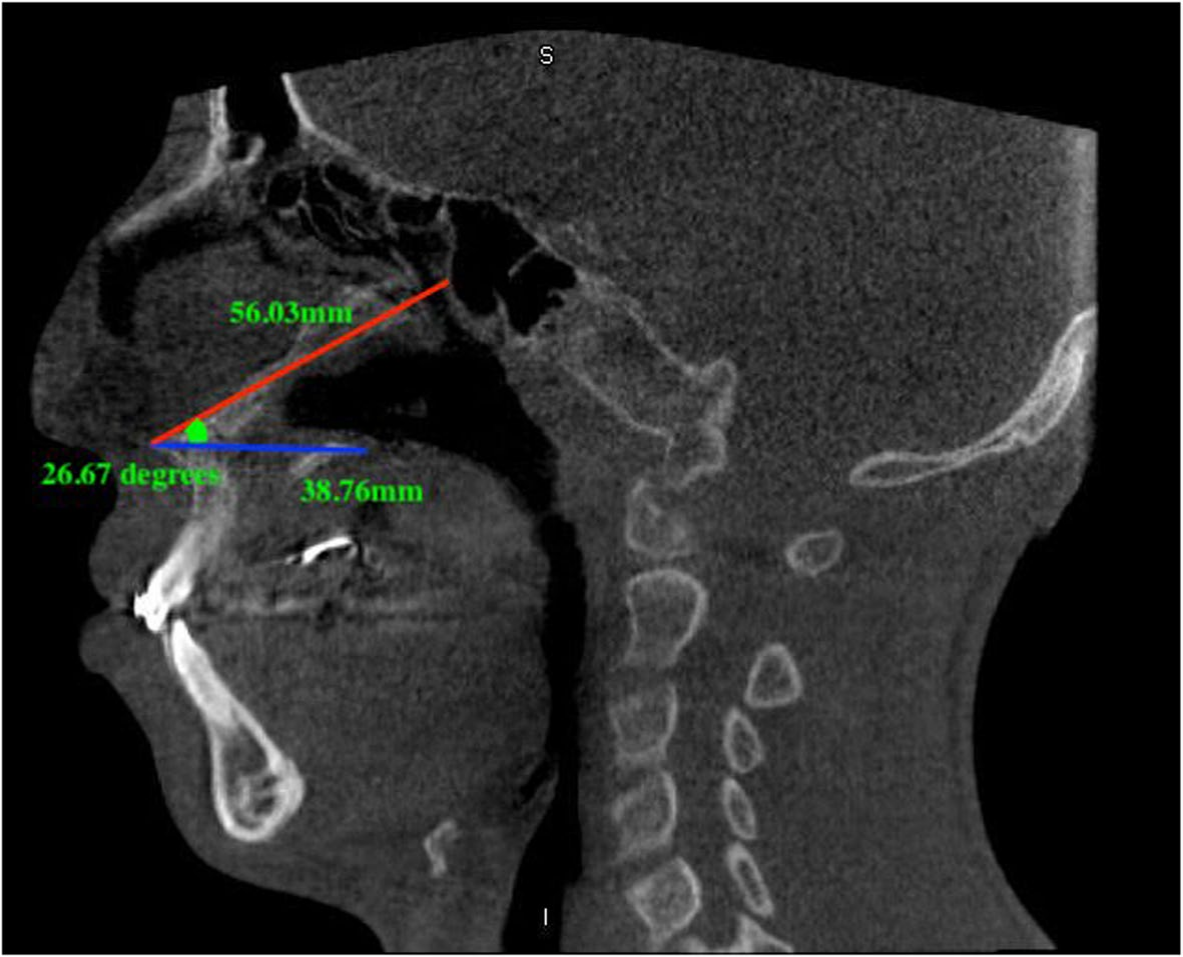
Length and angle measurements in CLP group

**Fig. 3:**
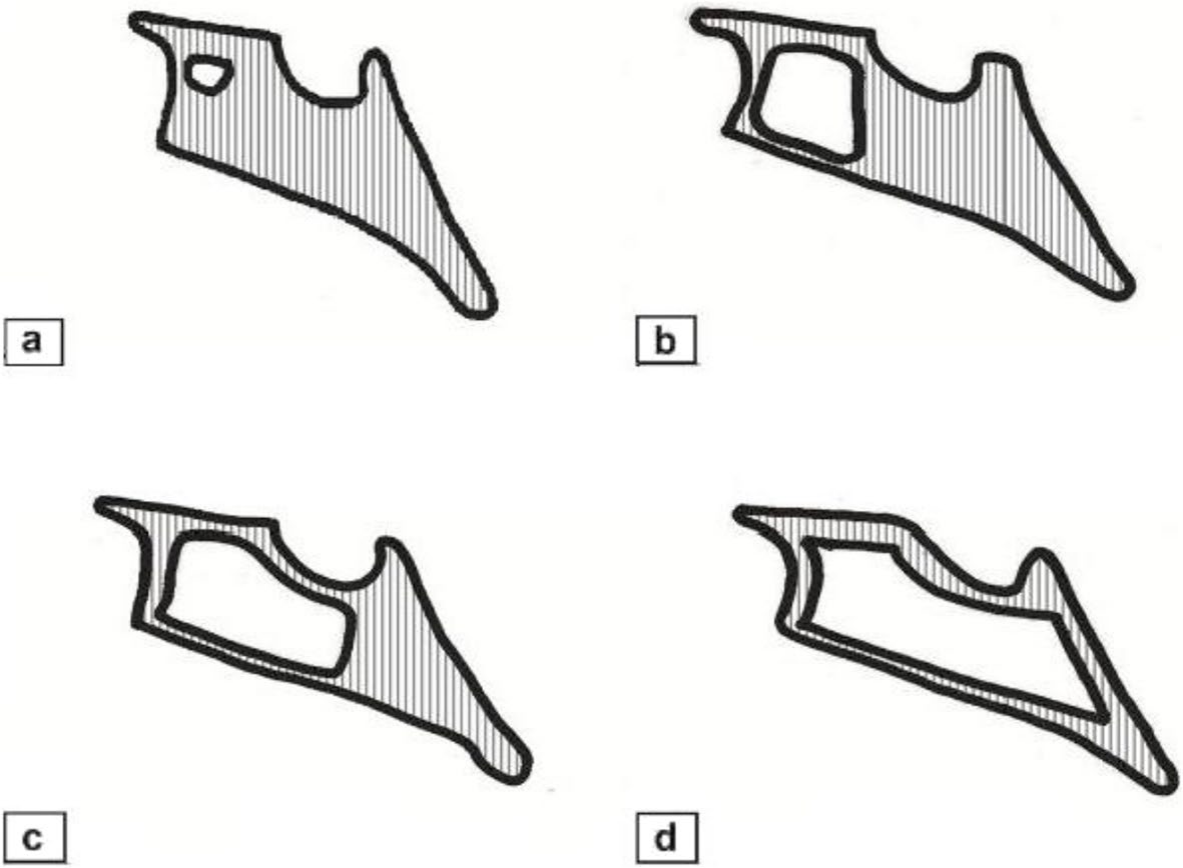
Types of Pneumatization; **a** conchal type, **b** presellar type, **c** sellar type, **d** postsellar type

Three values (two lines and one angle) were measured as follows: the surgical osteotomy line extended between the spina nasalis anterior point and the junction of the lower point of the perpendicular layer of the palatine bone, the undesired osteotomy line extended between the spina nasalis anterior and the lower anterior edge of the sphenoid sinus floor and the angle was between these two lines (Figs. 1 and 2). Pneumatization of the sphenoid sinus is divided into 4 groups as concal, presellar, sellar and postsellar [10]. In addition, sphenoid sinus types were examined in the CLP and control groups in our study and it was evaluated whether there was a difference between them (Fig. 3).

### Statistical analysis

For the statistical analysis, the IBM SPSS Statistics Version 20 package program (IBM Corp.; Armonk, NY) was used. When effect size 1 (large effect size), *P* < 0.05 and power (1 − ß) 0.80 was defined, *n* = 20 in any group was predicted. A Shapiro–Wilk’s test was used for validating the normality of the distributions, and an independent t-test was used to compare variables between the groups. The test results were considered statistically significant if *p* was < 0.05.

## Results

The age range of the individuals was between 14 and 35 (mean 18.87 ± 4.51). 20 (50%) of the individuals were male and 20 (50%) were female. The age distribution was similar across groups and genders, with no statistically significant differences found (Table 1; *p* > 0.05). In the CLP group, 14 (70%) presented with unilateral CLP and 6 (30%) with bilateral CLP. The sphenoid sinus types in the CLP and control groups are shown in Table 2. In both CLP patients and control subjects, the most common type of sphenoid sinus observed was the sellar type, while the conchal type of sphenoid sinus was not seen in either group. The surgical osteotomy line, the undesired osteotomy line and the angle values between them are shown in Table 3. Surgical line in CLP patients (mean 43.38 ± 4.79) and undesired line potentially leading to complications (mean 55.02 ± 3.24) were found to be significantly lower compared to the control group (mean 51.91 ± 4.32 and mean 59.14 ± 5.08, respectively) (*p* < 0.05). However, there was no statistically significant difference found in the angles between the two osteotomies in both the CLP and control groups (*p* > 0.05). This suggests that in CLP patients, the maxilla is smaller in the anteroposterior direction compared to control patients. However, the angle between them did not show a significant difference related to vertical growth pattern.

**Table 1:** Demographic statistics

Groups	Gender	N	Min. (Age)	Max (Age)	Mean (Age)	Std	*p*	
**CLP**	**Female**	11 (55%)	14	25	17.82	3.71	0.529	0.558
	**Male**	9 (45%)	14	33	19.22	5.99		
**Control**	**Female**	9 (45%)	16	23	18.11	2.20	0.275	
	**Male**	11 (55%)	16	35	20.27	5.38		

*Std* Standart Deviation

*Min* Minimum

*Max* Maximum

Statistically significant difference *p* < 0.05

**Table 2:** Distribution of patients according to sphenoid sinus types

Sphenoid Sinus Type	CLP Group (n)	Control Group (n)	Total n (%)
Choncal	0 (0%)	0 (0%)	0 (%0)
Presellar	2 (10%)	1 (5%)	3 (7.5%)
Sellar	11 (55%)	10 (50%)	21 (52.5%)
Postsellar	7 (35%)	9 (45%)	16 (40%)
Total n (%)	20 (100%)	20 (100%)

**Table 3:** Comparison of line measurement values and angle between CLP and control subjects

	CLP Group				Control Group				
	Min	Max	Mean	Std	Min	Max	Mean	Std	*p*
**Surgical Line(mm)**	34.53	51.16	43.38	4.79	44.11	61.14	51.91	4.32	0*
**Undesired Line(mm)**	46.86	61.35	55.02	3.24	52.48	69.58	59.14	5.08	0.004*
**Angle(°)**	17.60	28.81	22.60	2.81	18.22	27.27	22.66	2.55	0.942

^*^Statistically significant difference *p* < 0.05

## Discussion

Orthognathic surgery is usually the last step of treatment in the functional rehabilitation of patients with CLP, and maxillary osteotomy is most frequently performed in these patients due to the prevalence of hypoplastic maxilla. General treatment principles for orthognathic surgery for cleft lip and palate are very similar to those applied when performing surgery in patients without clefts [11]. Treatment planning and operation of orthognathic surgery in patients with cleft lip and palate are more difficult than in patients without cleft due to further advancement, inadequate tissue quality and worsening dental occlusion [12].

The number of patients with CLP who need orthognathic surgery for maxillary advancement varies greatly in the studies [3, 13–15]. The orthognathic surgery is needed in 25% of CLP patients to achieve the harmonious interrelation of the jaws and adequate facial aesthetics [3, 11].

Le Fort I osteotomy is the most commonly used method in the treatment of CLP with orthognathic surgery but it has many complication risks. In patients with cleft lip and palate, shorter maxilla can be seen due to iatrogenic scar tissue and interruption of the septovomerine suture. This causes a decrease in the margin of safety during osteotomy and increases the risk of damage. In a review study, it was stated that complications occurred 12.76% and the most common complication among these complications is failure in closing the pre-existing palatinal fistula (28.57%), followed by failure in closing the velopharyngeal failure (16.79%), pre-existing alveolar fistula closure (10.74%), gingival recession (4.55%), failure of the stabilization of the premaxilla in the bilateral CLP cases (4.55%) [16]. In another study, anatomical complications were reported 2.6%, deviation of nasal septum in 16 patients (1.6%), and nonunion of the osteotomy gap in 10 patients (1%). It was stated that 11 (1.1%) of patients with bimaxillary orthognathic surgery had severe bleeding requiring blood transfusion, and 1 patient (0.1%) needed that external carotid artery should be ligated. It was stated that 11 patients (1.1%) had significant infections such as abscess or maxillary sinusitis and none of them had osteomyelitis of all patients’ ischemic complications were seen in 10 patients (1.0%), and 2 of them (0.2%) reported that alveolar process had aseptic necrosis and 8 of these patients (0.8%) had gingival recession. In 5 patients (0.5%) it was reported that insufficient fixation of osteosynthesis material was observed [17]. In addition, oro-nasal fistula, alveolar and palatal cleft may also compromise vascular supply to the maxilla. Under these pre-existing variations, operative procedures should be modified for mucosal incision design, osteotomy line design, and intraoperative manipulation for lesser segments [18, 19]. Two- and three-part LeFort I osteotomies may be indicated to provide alveolar continuity and simultaneous closure of the oronasal fistula, especially in patients with alveolar and cleft palate [20]. Among all these complications, sphenoid sinus complications also take a serious place. In a case report, it was stated that blindness occurred in the right eye of the patient as a result of fracture in the ala major of the sphenoid bone and the anterior and lateral wall of the sphenoid sinus after Le Fort I surgery [21]. In another study, a fracture extending to the right sphenoid sinus sidewall occurred during the pterygomaxillary separation and the sphenoid sinus was filled with blood; accordingly, it was reported that late-onset nervus abducens palsy was observed [22]. Cleft lip and palate patients, in particular, can have anatomical variations because of previous palate surgery. The pterygomaxillary junction can be more sclerotic and there is a possibility that maxillary separation is more likely to occur close to the skull base or a fracture line can extend towards the skull base in these patients. Due to these variations, sphenoid injuries from osteotomy may occur in patients with cleft lip and palate [23]. Kim et al.[24] reported in a case report that there was injury to the sphenoid bone after osteotomy applied to a patient with cleft lip and palate. He strongly recommended to be very careful while separating the pterygomaxillary junction, especially in patients with cleft palate. Kim et al. reported that studies with three-dimensional quantitative analysis of the preoperative pterygomaxillary junction area are needed to confirm the anatomical variations in cleft patients.

The sphenoid sinus reaches adult size at age 14 [25]. Sella tursica stands out on the roof of the well-pneumatized sphenoid sinus and is known as the sellar process [26]. This is considered one of the most important surgical sites of the sellar plane [27]. Sphenoid sinus pneumatization is important in Le Fort I surgery. Pneumatization of the sphenoid sinus is classified into 4 groups: conchal, presellar, sellar and postsellar [10]. In a study, it was noted that 2% conchal, 24% were presellar, 41% were sellar and 33% were postsellar in total sinuses of 51 skulls [28]. In our study, sellar type sphenoid sinus 21 (52.5%), postsellar type sphenoid sinus 16 (40%), presellar type sphenoid sinus 3 (7.5%) were found. The conchal type sphenoid sinus was not detected in our study. In CLP group, %10 presellar type, %55 sellar type and %35 postsellar type sphenoid sinus were found.

Due to the severity of sphenoid sinus complications, there are also studies focusing on nasal septum osteotomy during Le Fort I surgery. In an anatomical study, Sareen et al. [29] evaluated the surgical anatomical features of the sphenoid sinus and they stated that the distance of the anterior nasal spina to the infero-anterior wall of the sphenoid sinus is 72 mm to 93 mm (average 79 mm). In another study, it was stated that the distance between the anterior nasal spine and sphenoid sinus 40.4 to 70.9 mm (mean 58.3 ± 5.9 mm) in men and whereas 45.0 to 63.2 mm in women (mean 55.2 ± 4.3 mm) and between the genders there was no statistically significant difference [30]. Saruhan et al. [31] studied on the CBCT sections of young adult patients and they stated that nasal septum osteotomies can be performed posteriorly an average of 50.64 mm in females, average of 52.51 mm in males and, undesired line is 62.76 mm in females and 64.25 mm in males. Also it has been stated that it should not be angulated towards the superior more than average of 16.80 degrees in females and 18.50 degrees in males. In our study, in control group surgical line was 44.11–61.14 mm (mean 51.91 ± 4.32 mm); undesired line was 52.48–69.58 mm (average 59.14 ± 5.08 mm); the angle was 18.22–27.27° (average 22.66 ± 2.55°). When this study and our study were compared in terms of surgical and undesired line values, it was seen that the results of the studies were similar. However, when compared in terms of angle, it was seen that the angle values were higher in our study in control group. Also in our CLP group, surgical line was 34.53–51.16 mm (mean 43.38 ± 4.79 mm); undesired line was 46.86–61.35 mm (average 55.02 ± 3.24 mm); the angle was as 17.60–28.81° (average 22.60 ± 2.81°). It was found that the surgical and undesired lines were lower in the CLP group than in the control group and statistically significant difference was found. When evaluated in terms of angle, it was seen that there was no statistically significant difference between the control group and the CLP group. We thought that the surgical and undesired line values ​​were lower in the CLP group because CLP reduced the development of the maxilla in the anterior posterior direction. But the angle to the sphenoid sinus was not affected by CLP. The results we found in our study concluded that less osteotomy is required in the perioperative surgical procedure due to the shorter distance from the anterior nasal spine to the lower anterior wall of the sphenoid sinus, and the distance from the anterior nasal spine to the perpendicular lamina. The angle measured and the osteotome position before the operation are important in terms of giving an idea to the physician. Thus, sphenoid sinus injuries due to incorrect angulation will be prevented.

Our study is the first study to compare the safety margin distance during nasal septum osteotomy according to the sphenoid sinus in CLP individuals. However, since the number of individuals was not sufficient, CLP individuals could not be evaluated separetly unilateral or bilateral CLP. In addition, lines and angle evaluation could not be made according to sphenoid sinus and unilateral and bilateral cleft types. These situations constitute the limitation of our study.

## Limitation

The study's limitations include a relatively small sample size, comprising 20 individuals with cleft lip and palate (CLP) and 20 controls, which may limit the generalizability of the findings to a broader population of CLP patients. The study did not differentiate between unilateral and bilateral CLP cases, and the analysis did not account for potential variations in surgical safety margins between these different CLP subtypes. This lack of subgroup analysis may overlook specific considerations needed for each type of CLP. Additionally, the study did not assess the impact of other potential factors, such as the severity of CLP or variations in surgical techniques, which could influence the safety margin during nasal septum osteotomy. These unexplored factors contribute to the complexity of the surgical procedure and may affect the generalizability of the study's conclusions. Its obvious and quite known that the growth of maxillary bone infleunces on the shape, size, position and degree of angulation of the nasal septum.

## Conclusion

Due to mid-facial and maxillary growth deficiency in individuals with CLP, the length of a nasal septum osteotomy on posterior direction should be planned to be less than in healthy individuals. On the other hand, there is an average of only 0.06° difference between healthy and CLP patients; therefore, planning need not take into account any difference in angulation between healthy and CLP patients.

### Supplementary Information

Supplementary Material 1.

## Data Availability

The datasets used and/or analysed during the current study are available from the corresponding author on reasonable request.

## Abbrevations

CLP: Cleft Lip and Palate
CBCT: Cone Beam Computed Tomography
FH: Frankfort Horizontal
